# Zika Virus Disease in Traveler Returning from Vietnam to Israel

**DOI:** 10.3201/eid2208.160480

**Published:** 2016-08

**Authors:** Eyal Meltzer, Yaniv Lustig, Eyal Leshem, Ran Levy, Giora Gottesman, Rotem Weissmann, Duha Hejleh Rabi, Musa Hindiyeh, Ravit Koren, Ella Mendelson, Eli Schwartz

**Affiliations:** Sheba Medical Center, Tel Hashomer, Israel (E. Meltzer, Y. Lustig, E. Leshem, M. Hindiyeh, R. Koren, E. Mendelson, E. Schwartz);; Tel Aviv University, Tel Aviv, Israel (E. Meltzer, E. Leshem, G. Gottesman, M. Hindiyeh, E. Mendelson, E. Schwartz);; Maccabi Health Services, Kfar-Yona, Israel (R. Levy);; Meir Medical Center, Kfar Saba, Israel (G. Gottesman, R. Weissmann, D. Hejleh Rabi)

**Keywords:** Zika virus, travel, Vietnam, Mexico, Dominican Republic, Colombia, Israel, viruses

**To the Editor**: On February 1, 2016, the World Health Organization designated the Zika virus disease outbreak in Latin America as a Public Health Emergency of International Concern (*1*). Genetic and epidemiological data suggest that Zika virus had been present in Southeast Asia since the 1940s (*2*); however, the disease burden and geographic extent of Zika virus disease in Asia are not clear. Occasional cases in some Asian countries, mostly in returning travelers, have recently been documented (*3**–**5*); however, as of February 2016, none were in Vietnam.

During December 2015–February 2016, the National Center for Zoonotic Viruses (Tel Hashomer, Israel), diagnosed 8 cases of Zika virus disease in travelers returning to Israel. The Center is part of the Central Virology Laboratory of the Israel Ministry of Health and is the reference laboratory for the diagnosis of Zika, dengue, and chikungunya virus infections in Israel. During the same period, 4 cases of dengue and 1 of chikungunya were also diagnosed. Of the 8 cases of Zika virus disease, 7 were in patients returning from South and Central America and the Caribbean (Technical Appendix) and 1 was in a patient returning from Vietnam via Hong Kong. We report the patient returning from Vietnam.

The patient was a 61-year-old man from Israel who spent 10 days in Vietnam during December 2015: 3 days in Hội-An, 3 in Hue, and 4 in Ho Chi Minh City. After spending 2 more days in Hong Kong, he returned to Israel. On the third day after his return, he experienced fever, malaise, and headache; he had no rash or conjunctivitis. Laboratory studies showed only lymphopenia and mildly elevated liver enzymes. Symptoms continued for 8 days and then resolved completely. His illness was initially suspected to be dengue; however, test results for dengue (NS1 early antigen, dengue capture IgM, and dengue IgG indirect; all 3 from Panbio, Brisbane, Queensland, Australia) and chikungunya (Anti-Chikungunya Virus IIFT; Euroimmun AG, Lübeck, Germany) viruses were negative.

In Israel, Zika virus diagnostic tests were introduced in December 2015 and are available only through the National Center for Zoonotic Viruses. Serologic testing for Zika virus is performed by using an ELISA IgM and IgG kit (Euroimmun AG), which detects antibodies against the Zika nonstructural protein NS1 and is therefore considered very specific for Zika virus infection (*6*). Zika real-time reverse transcriptase PCR (rRT-PCR) against part of the envelope gene (1086–1162 bp) was adopted from the method established during the Zika virus outbreak in Micronesia (*7*).

In the traveler to Vietnam, rRT-PCR and serologic results were positive for Zika virus RNA and antibodies, respectively. For sequencing of Zika virus RNA, we amplified a 327-fragment from the prM and envelope genes by rRT-PCR, using primers Zika virus 835 (5′-TTGGTCATGATACTGCTGATTGC-3′) and Zika virus 1162c (5′-CCACTAACGTTCTTTTGCAGACAT-3′) and an ABI 3500 Genetic Analyzer (Applied Biosystems, Foster City, CA, USA). A Bayesian maximum clade credibility time-scaled phylogenetic tree (BEAST, http://beast.bio.ed.ac.uk/Main_Page) of the 231-nt fragment obtained from this patient and from 3 other patients from Israel who acquired their infection in South and Central America (Technical Appendix) was performed with 19 reference Zika virus strains. To infer the evolutionary relationships and the most recent common ancestor for the Zika virus fragment of the envelope gene, we applied the Bayesian Markov chain Monte Carlo method by using a relaxed molecular clock, as implemented in BEAST version 1.7.5. Trees were visualized and edited with FigTree version 1.4.2 (included in BEAST software). Altogether, the analysis showed that the virus belonged to the Asia Zika virus lineage and seems to be highly similar to strains currently circulating in Latin America (Figure). However, sequencing of a larger segment would be needed for a more accurate phylogency.

**Figure:**
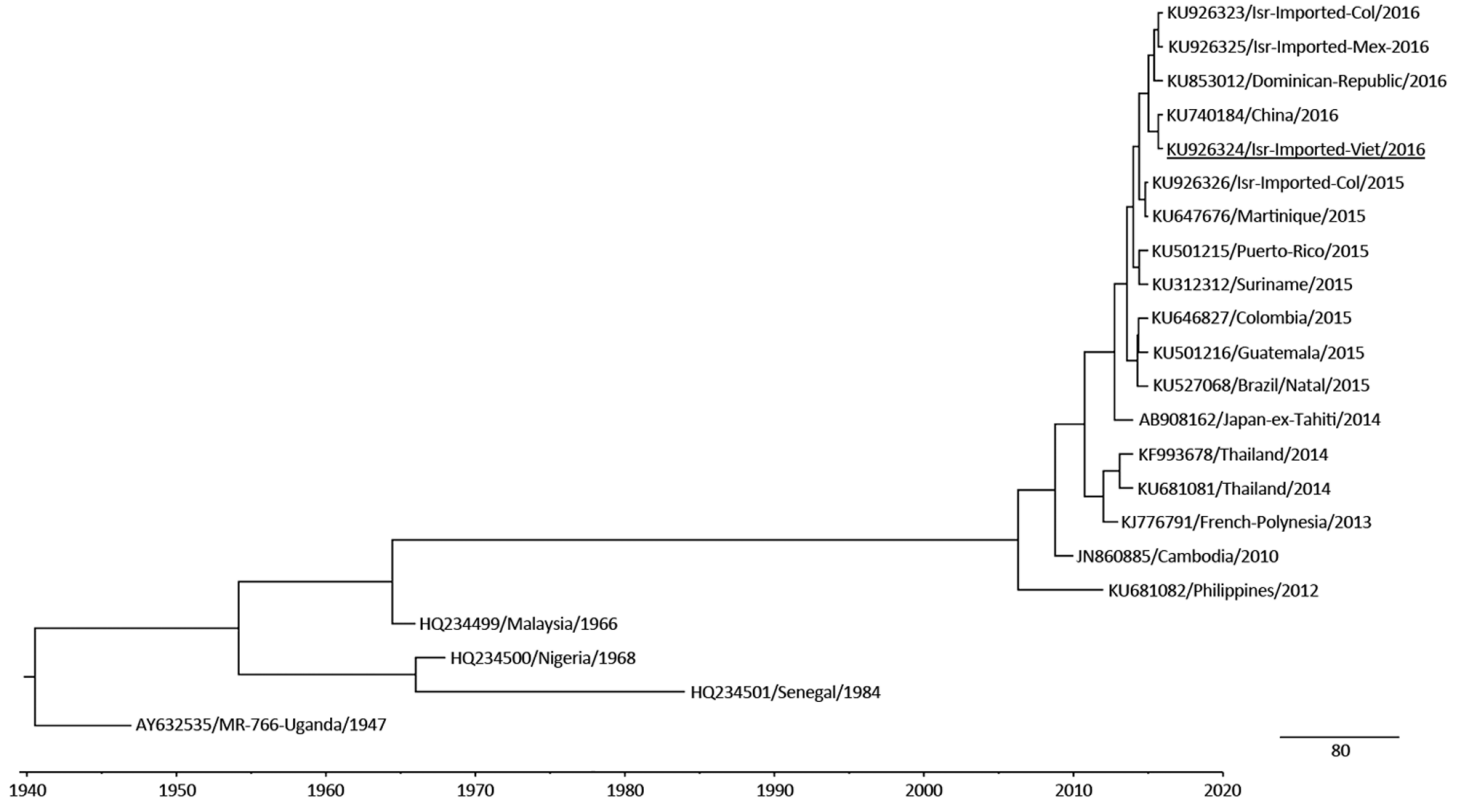
Phylogenetic tree of Zika virus RNA isolated from travelers returning to Israel. Bayesian maximum clade credibility time-scaled phylogenetic tree (BEAST, http://beast.bio.ed.ac.uk/Main_Page) was generated by using 4 partially sequenced Zika virus envelope genes (231 bp) detected from 4 samples obtained from patients in Israel during 2015–2016 and 19 reference strains belonging to the lineages from Asia and Africa. Isr, Israel; Viet, Vietnam; Col, Colombia; Mex, Mexico. Underlining indicates Zika virus imported from Vietnam. Scale bars indicate units in time (years).

This case illustrates the role of returning travelers as potential disease sentinels and the inadequacy of information about Zika virus circulation in Asia. During December 2015–January 2016, when this patient was evaluated and followed up, no cases of Zika virus disease had yet been reported from Vietnam. Since then, a case in a traveler from Australia has been reported (*8*). In addition, in late March 2016, health authorities in Vietnam reported 2 autochthonous Zika virus cases in women from Nha-Trang and Ho Chi Minh City (*9*). Because the incubation period for Zika virus is not clearly defined, we are unable to definitely rule out Hong Kong as the source of infection. However, to our knowledge, Zika virus circulation in Hong Kong has not yet been reported. Assuming the most probable incubation period to be 5–8 days, we believe that the patient who visited Vietnam most likely became infected with Zika virus in Ho Chi Minh City.

Until more thorough epidemiologic data from Asia become available, testing all travelers returning from Southeast Asia with exanthema, fever, or other signs or symptoms suggestive of Zika virus disease is justified. In addition, because during this period Zika virus had become the most frequent arbovirus isolated from travelers returning to Israel, Zika virus now seems to be a substantial cause of febrile illness in travelers returning from Zika virus–endemic regions.

Technical Appendix Epidemiology and diagnosis of Zika virus disease and case descriptions for travelers returning to Israel during December 2015–February 2016.
