# Supplementary material for: Zika Virus Disease in Traveler Returning from Vietnam to Israel
Source: Emerg Infect Dis. 2016 Aug;22(8):1521–2. doi: 10.3201/eid2208.160480 (PMC4982162; doi:10.3201/eid2208.160480)
Supplement: Technical Appendix — Epidemiology and diagnosis of Zika virus disease and case descriptions for travelers returning to Israel during December 2015–February 2016. [file 16-0480-Techapp-s1.pdf]

Article DOI: http://dx.doi.org/10.3201/eid2208.160480

Zika Virus Disease in Travelers Returning from Vietnam to Israel

Technical Appendix

Case Descriptions

Colombia: An Israeli family of four: 37 years old male, 31 years old female and two daughters aged 8 and 2 years, all previously healthy, had traveled to Colombia for seventeen days during December 2015. Their trip included, in addition to Bogota and Subachoque (elevation ≈2600 m), 5 days in Girardot: a low-lying city in central Colombia (elevation 326 m). After returning to Bogota, all four had sequentially become ill with symptoms suggestive of ZIKV infection, which was laboratory-confirmed in three after their return. The virus was sequenced in one of the family members (Table).

A fifth Israeli traveler had traveled to Colombia from 27 January – 2 February 2016, where he stayed in Bogota, and for 5 days in La Dorada: a low-lying city on the Magdalena River (elevation 176 m). Three days after his return fever and conjunctivitis developed.

The Sixth ZIKV case from Colombia was of an Israeli woman, who had traveled for 25 days during November 2014 in South and Central America. After 10 days in Colombia, of which 8 were spent in areas below 2000 m (Cartagena), she was 2 days in Panama, and 4 days later (7 days after leaving Cartagena) while in Costa Rica she developed fever, rash and myalgia. Symptoms resolved while being treated with steroids for her rash, and she continued her trip in Costa Rica, where she had significant freshwater and jungle exposure, followed by 5 days in Cuba. She became ill again with fever and severe myalgia 3 days after her return to Israel, was treated for presumed leptospirosis and recovered. PCR and serology for ZIKV was positive, however, leptospirosis was also confirmed by seroconversion in convalescent serum. We concluded that ZIKV acquired in Colombia had in all likelihood caused the earlier, self-limited fever and exanthema, whereas leptospirosis acquired in Costa Rica caused the second febrile episode.

Mexico: A 29-year-old Israeli woman had traveled for 2 weeks in Guatemala, and then for 1 week in Mexico, where she stayed the whole period in Yucatan, with last 4 days in Cancun. Three days after her return fever developed, which lasted for 4 days.

Dominican Republic: A 29-year-old Israeli had traveled for 3 weeks during January 2016 in the Caribbean region: Cuba for 10 days, and the in the Dominican Republic, where he stayed in Santo Domingo, and for 5 days in Jaragua National Park on the coast. Three days after leaving the Dominican Republic fever and rash developed that lasted for 4 days.

Technical Appendix Table. Epidemiology and diagnosis of Zika virus in travelers returning to Israel, December 2015 through February 2016*

No.	Travel date	Age, y/sex	Probable country of exposure	Most likely place of exposure	Duration of exposure, d	Chikungunya IgM	Chikungunya IgG	Dengue NS1	Dengue IgM	Dengue IgG	Diagnostic method
1	Nov 2015	50/F	Colombia/ Panama	Cartagena†	8	Neg	Neg	Neg	Neg	Pos	PCR‡ and serology
2	Dec 2015	31/F	Colombia	Girardot§	5	Neg	Neg	Neg	Neg	Pos	PCR and serology
3	Dec 2015	37/M	Colombia	Girardot§	5	Neg	Neg	Neg	Neg	Pos	Serology
4	Dec 2015	2/F	Colombia	Girardot§	5	ND	ND	ND	ND	ND	PCR‡ (serology ND)
5	Dec 2015	61/M	Vietnam	Ho Chi Minh City, Hội-An, Hue	10	Neg	Neg	Neg	Neg	Neg	PCR‡ and serology
6	Jan 2016	29/M	Dominican Republic	Santo Domingo, Jaragua National Park¶	7	Neg	Neg	Neg	Neg	Pos	PCR and serology
7	Jan 2016	29/F	Guatemala/ Mexico	Cancun#	7	Neg	Neg	Neg	Neg	Neg	PCR‡
8	Jan–Feb 2016	30/M	Colombia	La -Dorada§	5	Neg	Neg	Neg	Neg	Neg	Serology

*ND, not performed; neg, negative; NS1, nonstructural protein 1; pos, positive.
†Most likely area of exposure designated assuming 5–8 d incubation period. Patient 1 had stayed 2 d in Panama after leaving Cartagena, Colombia. Symptoms began 2 d later, while she stayed in Costa Rica.
‡Virus isolates from these cases were sequenced.
§Sole area of potential exposure to Zika virus.
¶Patient 6 had traveled in Cuba (which had not reported autochthonous cases of Zika virus until March 2016) for 10 d, and to the Dominican Republic. Symptoms began 3 d after leaving the Dominican Republic (and 19 d after departing from Cuba).
#Patient 7 had traveled for 2 wk in Guatemala, and then for 1 wk in Mexico, Symptoms began 3 d after her return from Mexico (12 d after departing from Guatemala).
